# Optimization of the margin expanded from the clinical to the planned target volume during intensity-modulated radiotherapy for nasopharyngeal carcinoma

**DOI:** 10.18632/oncotarget.22518

**Published:** 2017-11-20

**Authors:** Wang Fangzheng, Sun Quanquan, Jiang Chuner, Ye Zhimin, Yang Shuangyan, Yu Huanhuan, Shi Jianfang, Masoto Sakamoto, Qin Weifeng, Fu Zhenfu, Jiang Yangming, Wang Yuezhen

**Affiliations:** ^1^ Department of Radiation Oncology, Zhejiang Cancer Hospital, Hangzhou 310022, China; ^2^ Radiobiology Research Unit Key Laboratory of Radiation Oncology of Zhejiang Province, Hangzhou 310022, China; ^3^ Department of Breast Surgery, Zhejiang Cancer Hospital, Hangzhou 310022, China; ^4^ Department of Physics, Zhejiang Cancer Hospital, Hangzhou 310022, China; ^5^ Department of Radiology, Fukui Red Cross Hospital, Fukui 918-8501, Japan; ^6^ Department of Digital Earth, Institute of Remote Sensing and Digital Earth, CAS, Beijing 100101, China

**Keywords:** nasopharyngeal carcinoma, intensity-modulated radiotherapy, margins, clinical target volume, megavoltage computed tomography

## Abstract

During the radiotherapy process, the emergence of set-up errors is nearly inevitable. Because set-up errors were not detected and corrected daily, planned target volumes were formed by expanding the clinical target volume according to each unit's experience. We optimized the margins of clinical and planned target volumes during administration of intensity-modulated radiotherapy for nasopharyngeal carcinoma. A total of 72 patients newly diagnosed with non-metastatic nasopharyngeal carcinoma and treated with Tomotherapy were prospectively enrolled in the study. For each patient, one megavoltage computed tomography scan was obtained after conventional positioning, online correction, and daily tomotherapy delivery. The interfraction set-up errors were determined using a planning CT based on the registered scan. The mean interfraction errors were -2.437±2.0529 mm, 0.0652±2.3844 mm, 0.318±1.8314 mm, and 0.197±1.8721° for the medial-lateral, superior-inferior, and anterior-posterior directions, and the direction of rotation, respectively. The total M_PTV_ in the three directions was 7.53 mm, 1.83 mm, and 2.08 mm, respectively. The 3-mm margins in the superior-inferior and anterior-posterior directions uniformly expanded from the clinical target volume should be sufficient, and the marging in the medial-lateral direction was up to 7.5 mm. These results suggest that personalized M_PTV_ may be adopted for intensity-modulated radiotherapy planning.

## INTRODUCTION

Nasopharyngeal carcinoma (NPC) is a common head and neck cancer in southern China [1, 2]. Due to the special anatomical structure and high radiosensitivity, radiotherapy has been as the preferred method of treating NPC. Intensity-modulated radiation therapy (IMRT) is a new approach that has been widely adopted for treatment of NPC due to its ability to provide more conformal dose distributions with sharp dose gradients and to spare the surrounding organs at risk (OARs) [3]. With use of IMRT for NPC, the beneficial effects of treatment were improved, while the toxicities were reduced [4, 5]. In the process of radiotherapy, the emergence of the setup errors is inevitable and underestimated. Guckenberger et al. [6] found that in patients with head-and-neck cancer, translational errors were ≥2 mm in 13.9% of all measurements for each axis, separately, and rotational errors were >2° in 11.1% of all measurements. Because setup errors were not detected and corrected daily, the planned target volume (PTV) was formed by expanding the clinical target volume (CTV) by a selected amount based on each unit's experience. The IMRT plan is designed in single computed tomography (CT) images obtained through CT simulation. The dose distribution in the target volume and normal tissues varies daily in the course of treatment, due to a variety of system and random errors [7]. Improving the positioning accuracy of IMRT is imperative.

Due to equipment and technical implementation differences, IMRT was divided into static IMRT, volumetric arc IMRT, and Tomotherapy IMRT [8–11]. Compared with the other two methods, Tomotherapy has a greater dosimetric advantage [11]. The fan-beam Megavoltage computed tomography (MVCT) carried out by itself could detect and correct the set-up errors online and daily to guarantee the accurate treatment of NPC treated with Tomotherapy. The position error was obtained using an electronic portal imaging device (EPID) and kilovoltage (KV) X-ray cone beam computed tomography (CBCT) [12–15]. There is still a certain gap due to the frequency of once per week. This study was conducted to assess the intrafractional errors in NPC patients treated with Tomotherapy through daily MVCT imaging, and to optimize the margin expanded from CTV to PTV.

## RESULTS

### Position errors

**Figure 1:**
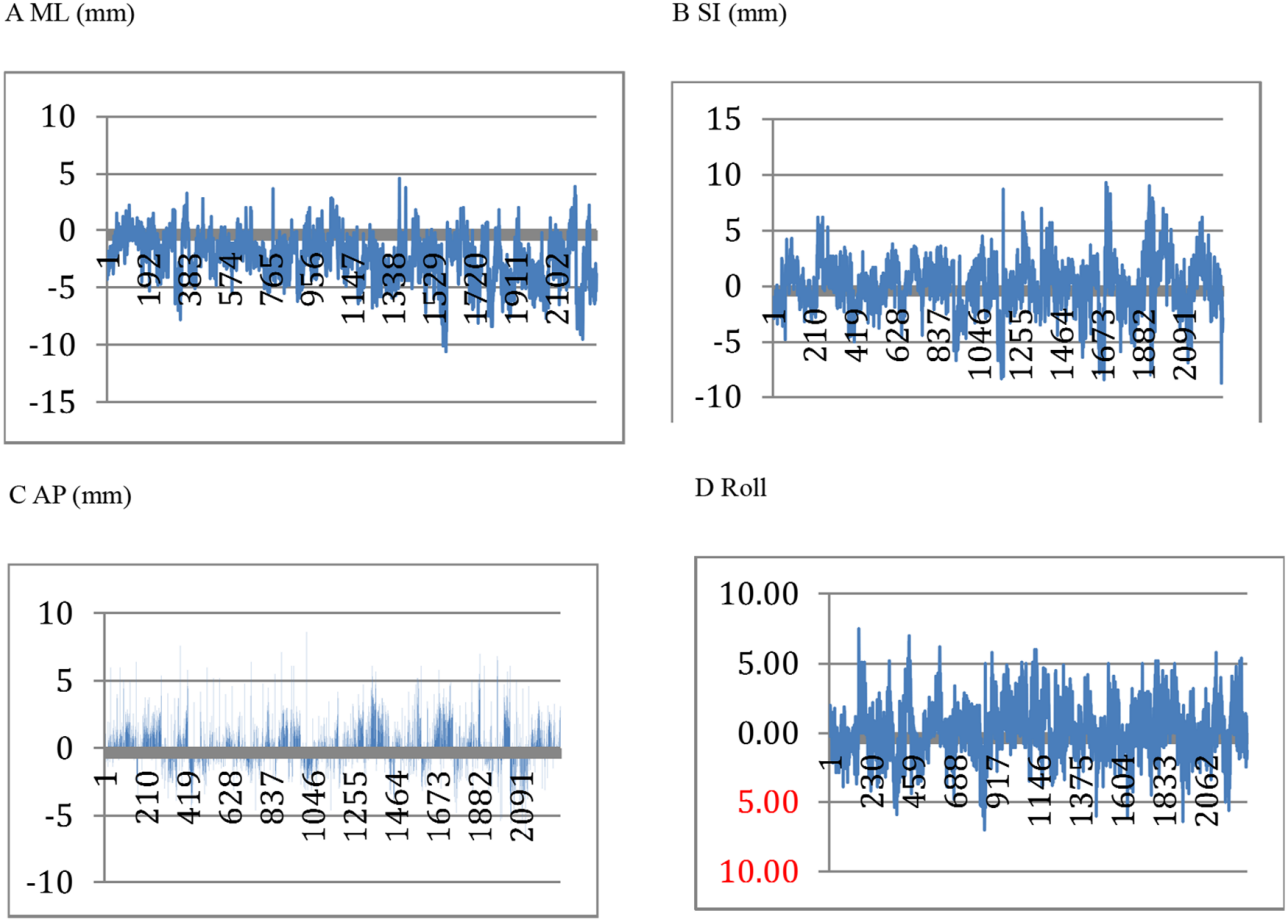
The distributions of the set-up errors in ML, SI, AP directions and Roll

2290 MVCT scans were collected from 72 patients. The mean value and standard deviation of the set-up errors were obtained in medial-lateral (ML; -2.437±2.0529 mm), superior-inferior (SI; 0.0652±2.3844 mm), and anterior-posterior (AP; 0.318±1.8314 mm) directions and rotation degrees (Roll; 0.197±1.8721°). The distributions of the set-up errors in each direction are shown in Figure 1.

**Figure 2:**
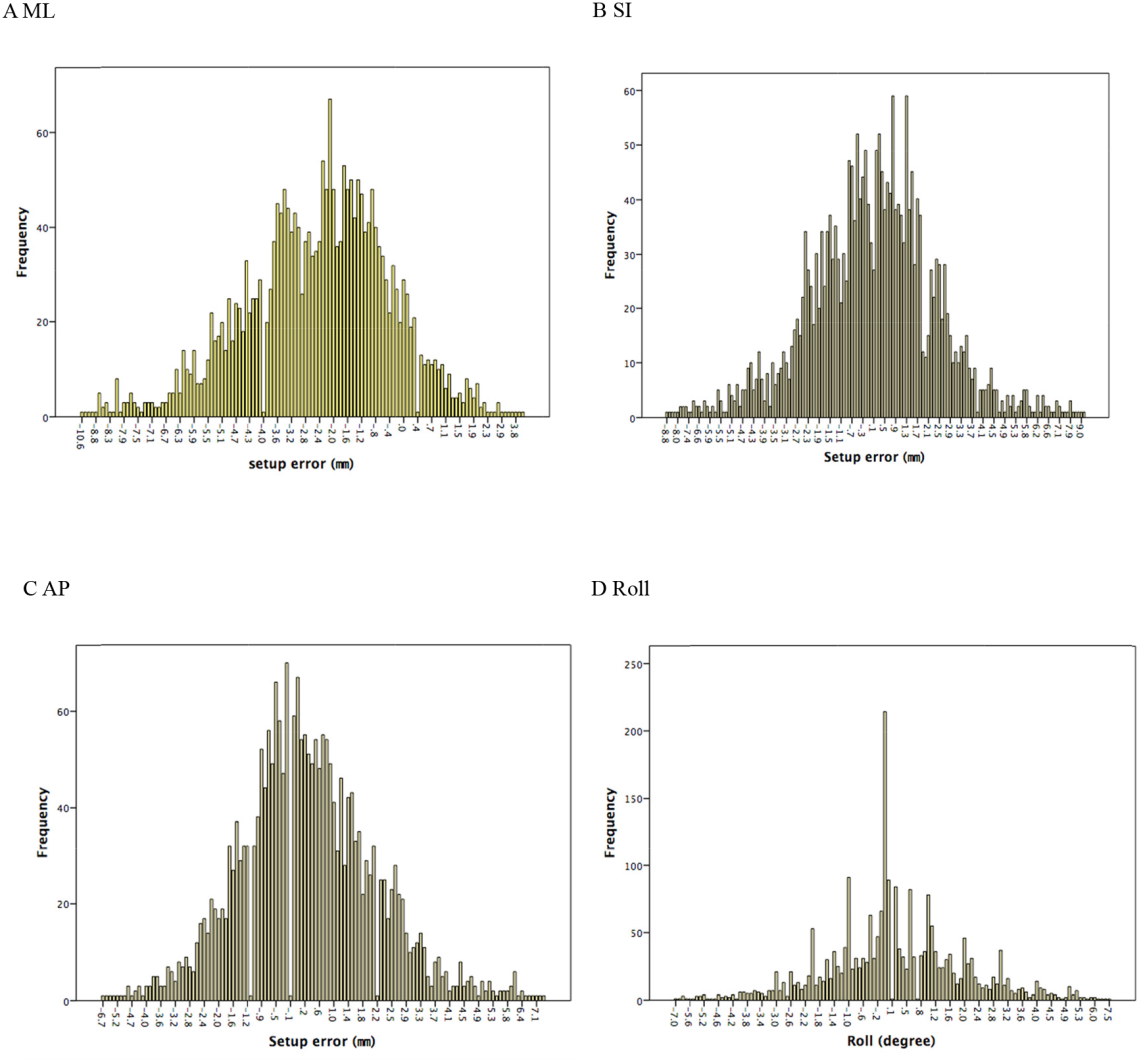
The histogram of the distribution frequency of the absolute values for the set-up errors in all directions

The numbers of the absolute errors greater than 3 mm in ML, SI, AP directions were 890 (38.9%), 400 (11.5%), and 233 (10.2%) times, respectively. The numbers of the set-up error more than 5 mm were 256 (11.2%), 114 (5%), and 43 (1.9%) times, respectively. The numbers of the rotation angle greater than 3° and 5° were 265 (11.5%) and 52 (2.3%) times, respectively. Figure 2 shows that the distribution frequency of the values for the set-up errors in all directions.

**Figure 3:**
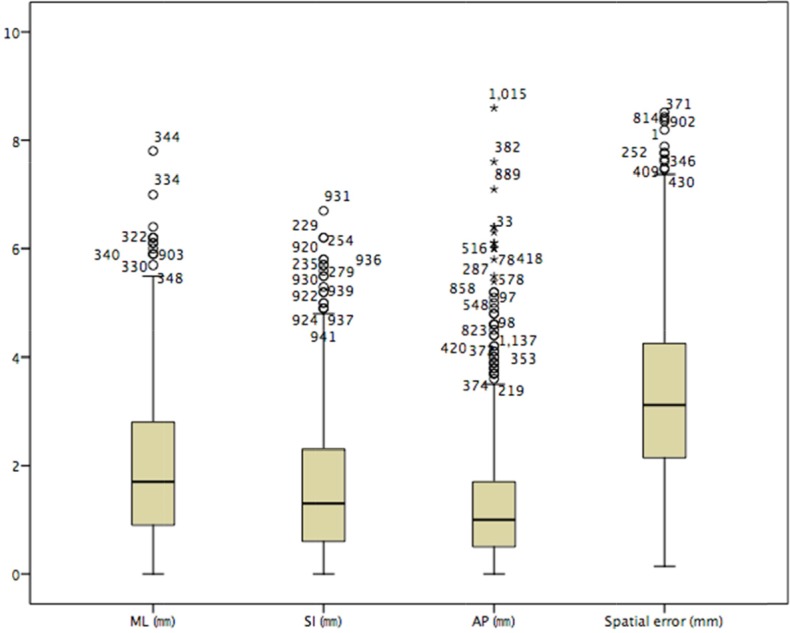
The box diagram of the absolute setup errors in the three-dimensional orientation

The mean value of the absolute set-up error and standard deviation were ML 1.953(± 0.0395) mm, SI 1.540(±0.0346) mm, AP 1.273(±0.0330) mm, and Roll 1.466(±0.0399)°, respectively. The mean value and standard deviation of the spatial displacement error were 3.2730(± 0.04366) mm (Figure 3).

The system errors and the random errors in the ML, SI, and AP direction are shown in Table 1. According to the margin formula recommended by Van-Herk [16], the values of MPTV were ML 7.53 mm, SI 1.83 mm, and AP 2.08 mm. Compared to the CTV-PTV margin of 3 mm, the margin was greater than 3 mm in the ML direction, while the margins of 3 mm in the SI and AP directions were sufficient. Therefore, whether the margin uniform expansion in each direction is suitable requires further study in clinical work.

**Table 1:** The estimation of the extended boundary in the direction for the patients with NPC (mm)

Direction	System error	Random error	M_PTV_
ML(mm)	2.437	2.0529	7.53
SI(mm)	0.0652	2.3844	1.83
AP(mm)	0.318	1.8314	2.08

## DISCUSSION

Radiotherapy is the main treatment for NPC and has been improved by the development of equipment with a radiation source of deep X-ray to cobalt-60, then to high-energy X-ray, and the treatment planning system (TPS) of manual calculation, 2D–3D TPS. IMRT is a substantial leap in radiotherapy for NPC. With the improvement of equipment and technology, radiation therapy aims to improve the local control and reduce the side effects. The 5-year overall survival rate increased from 20-30% in the early stage and 60-70% in the middle stage to 80-90% at present due to this process [17–19]. IMRT is the first choice of radiotherapy for NPC and is categorized into static IMRT, volumetric arc IMRT, and Tomotherapy IMRT [8–11]. The dosimetric optimization has been further improved [11]. Improving the accuracy of radiation therapy will improve the patient's fixed equipment and reduce the position error. At the same time, the experience of the definition for target volumes and the expanded margins, which originated from the two-dimensional radiation era, may be inconsistent with the requirements of precision treatment. Further investigation is required to stay consistent with the development of the technique.

In the early IMRT used for NPC, the set-up errors were obtained from EPID. The CTV-PTV margin reported was 5 mm [12, 13]. By the analysis of EPID images of 32 patients with NPC, the set-up errors were an X-axis error of 1.36(±1.02) mm, Y-axis error of 1.23(±1.05) mm, and a Z-axis error of 1.34 (±1.08)mm. The results suggest that the 5 mm margin of CTV-PTV was relatively safe [20]. The emergence of CBCT with more accuracy has replaced EPID [13]. Based on CBCT, the mean and standard deviation of the three dimensional directions were ML 0.180(±0.119) cm, SI 0.136(±0.112) cm, and AP 0.107(±0.084) cm [7]. Most of the results obtained in the literature show that the margin was 3 mm because the image sharpness was improved, but these data were collected once a week [14, 15].

Therefore, the dose deviation will increase further in the daily course of treatment, especially in the normal tissue. During the Tomotherapy process, the set-up error was corrected by MVCT image before each treatment to irradiate the tumor more precisely and protect the surrounding normal tissue, and the clinical results were confirmed [21].

The present study analyzed the set-up errors in the 2290 MVCT scans from 72 NPC patients treated with Tomotherapy. The mean value and standard deviation of the set-up errors were ML -2.437(±2.0529) mm; SI 0.0652(±2.3844) mm; AP 0.318(±1.8314) mm, and Roll 0.197(±1.8721)°. Wang et al. evaluated the set-up errors by using CBCT from 22 patients undergoing IMRT for NPC and found that the precorrection systematic errors ranged from 1.1–1.3 mm, and the random errors were also 1.1–1.3 mm [22]. In a study published in Journal of Practical Oncology [23], the set-up errors were analyzed weekly using the MVCT scanning technique in patients with head and neck cancer (HNC), thoracic cancer or abdominal cancer. In the 20 patients with HNC, the overall set-up errors were 1.93(±0.85) mm, 2.36(±1.25) mm, and 2.15(±1.52) mm in the RL, SI, and AP directions, respectively, before correction. Lu et al. conducted a prospective study to assess set-up errors during the treatment of IMRT by using daily CBCT. The overall set-up errors were 1.2(±1.0) mm, 0.8(±1.1) mm, and 1.7(±1.2) mm in the RL, SI, and AP directions, respectively [15]. However, van Kranen et al. found that local set-up errors were larger than the overall set-up error during the treatment, ranging from 1.1–3.4 mm (systematic) and 1.3–2.5 mm (random) [24]. Hurkmans et al. carried a review to assess the set-up verification in HNC patients using portal imaging, and concluded that Σ and σ varied by 1.6-4.6 mm and 1.1-2.5 mm, respectively [16]. Although our results were large, they were similar to the results obtained by van Kranen and Hurkmans.

Optimal CTV-PTV margins are important for local control of tumor and sparing the normal tissue. van Asselen et al. illustrated that reducing the margins of CTV and PTV improved parotid sparing and decreased the other complications [25]. Wang et al. showed that the PTV margins for precorrection, pretreatment, and posttreatment positions were 3.5–4.2 mm, 1.6–1.8 mm, and 2.5–3.2 mm, respectively [22]. Dionisi et al. analyzed the local positioning error of 44 HNC patients with CBCT, and found that PTV margins were 3.48 mm, 4.08 mm, and 4.33 mm in RL, SI, and AP directions, respectively, before correction [26]. Lu et al. found that a margin of 4.9 mm, 4.0 mm and 6.3 mm was required in the RL, SI and AP directions, respectively, and posited that a margin of 4-6.3 mm is required to ensure adequate coverage of the CTV when daily CBCT corrections are not performed [15]. A margin of 3-5mm was recommended for application in clinical practice. However, the application of narrow margins must be based on the premise of excellent quality-control measures such as daily MVCT online correction. In the current study, the margin in the ML direction was up to 7 mm, which more than that in previous studies. The 3 mm margin uniformly expanded from CTV was not appropriate because the distance in the ML direction was up to 7.53mm. If we designed PTV for IMRT according to the 3 mm margin uniformly, part of the tumor only received low-dose irradiation. Our results provide a theoretical basis for individualized margins from CTV to PTV. In the future, we recommend 3 mm margins in SI and AP directions, and 5 mm in the ML direction for NPC patients receiving IMRT without online correction, and a margin of 2-3 mm for those treated with Tomotherapy and daily MVCT.

## MATERIALS AND METHODS

### General information

72 patients with histologically proven NPC and treated with Tomotherapy from February 2015 to September 2016 in the Zhejiang province Cancer Hospital were enrolled. They had no distant metastasis. There were 55 males and 17 females with a sex ratio of 3.24:1. The median age was 56 years (18-70 years). According to the pathological classification [27], all patients had nonkeratinizing carcinoma. They were staged according to the 7th edition of International Union Against Cancer (UICC)/American Joint Committee on Cancer (AJCC) (2010) TNM staging system [28]: 6 patients in stage II, 28 stage III, and 38 in stage IV.

### Radiation therapy

All patients were immobilized in a supine position with the head in a neutral position with a tailored thermoplastic mask (MED-TEC Industries, USA) covering the head, neck, and shoulders. Intravenous contrast-enhanced CT using a 2-mm slice from the vertex to the manubriosternal joint was performed on a GE Spiral CT simulator for planning. The scanning images were transferred to RayStation (RaySearch Laboratories AB) through DICOM networks, target definition was referred to from the International Commission on Radiation Units and Measurements (ICRU) 50, ICRU62 [29, 30]. The delineation of NPC target volumes during the IMRT treatment was performed as described previously [31–34]. Gross tumor volume (GTV) referred to the tumor extent found in clinical and imaging examinations, including primary tumor (GTVnx) and metastatic lymph nodes (GTVnd). The high-risk clinical target volume (CTVnx) included GTV plus a 7 mm margin and encompassed the entire nasopharyngeal mucosa plus 5 mm submucosal volume. CTV1 was designed for potentially involved regions and included the whole nasopharyngeal cavity, the anterior 1/3—2/3 of the clivus (when invaded, the whole clivus should be covered), the skull base, the pterygoid plates, the parapharyngeal space, the inferior sphenoid sinus (the whole sphenoid sinus should be covered for stages T3 and T4), the posterior 1/4—1/3 of the nasal cavity, and the maxillary sinus. Level Ib was considered high risk in patients with metastatic lymph nodes in level IIa, and any lymph node drainage pathways containing metastatic lymph nodes were considered high risk. Low-risk CTV2 referred to levels IV and Vb without metastatic cervical lymph nodes. The planning target volume (PTV) was created based on each volume with an additional 3 mm margin, allowing for set-up variability. Critical normal structures, including the brainstem, spinal cord, parotid glands, optic nerves, chiasm, lens, eyeballs, temporal lobes, temporomandibular joints, mandible, and hypophysis were contoured and set as OARs during optimization.

The treatment was performed with a simultaneous integrated boost technique, using 6 MV photons. The prescribed radiation dose was 66 or 70.5 Gy to PGTVnx, 66-69 Gy to PGTVnd, 63-66 Gy to PTVnx, 60-63 Gy to PTV1, and 51-54 Gy to PTV2, delivered in 30 or 33 fractions. Radiation was delivered once daily, five fractions per week, over 6 -6.5weeks. The volume of PTV encompassed by less than 95% of the prescription dose should not exceed 1%. More than 110% of the prescription dose was not allowed in or out of PTV. The dose to OAR was limited by the RTOG 0225 protocol. Tomotherapy plans were designed by the TomoHTM Version 2.0.5 planning system.

### Chemotherapy

The NPC patients with III and IV stage received platinum-based induced chemotherapy for 2-4 cycles and platinum-based concurrent chemotherapy for 2 cycles during Tomotherapy, and 14 patients were treated with 6-8 cycles nimotuzumab.

### Image acquisition and registration

The image acquisition was obtained from the fan-beam MVCT scanning system carried by the accelerator. The patients with NPC received daily routine placement, MVCT scanning, and CT image registration before performing treatment of Tomotherapy each day. Then the accelerator software was completed to register the region of interest including the tumor, bone tissue, and important organs around the tumor, and calculated the set-up errors in the center about medial-lateral (ML), superior-inferior (SI), anterior-posterior (AP) and the direction of rotation. We compared the set-up errors of three dimensions such as ML, SI, AP and Roll between the MVCT and planning CT.

### The data analysis of the set-up error and the calculation of the range externally expanded

The set-up errors of the ML, SI, AP direction and the rotation angle were recorded, then the mean absolute error and standard deviation were calculated. The three-dimensional displacement value was calculated by the formula [35] F=(X^2^+Y^2^+Z^2^)^1/2^ (F represents the three-dimensional displacement error, X represents the value of ML direction, Y represents the value of SI direction, Z represents the value of AP direction). The average error value, system error, and random error of all the patients were computed. The error of M was calculated according to the formula proposed by VanHerk [16] (MPTV = 2.5Σ+ 0.7б, guarantee 95% of the prescription dose of at least 90% patients). According to the Stroom definition of the error estimation [36]: the mean value of earch patient's position error is individual systematic errors, and the standard deviation of each patient's position error is individual random errors; while group systematic errors (Σ) is the standard deviation of the individual systematic errors, and random errors (σ) is the standard deviation of the individual random errors. The formula for calculating the set-up error boundary of rotation was not designed at home and abroad.

### Statistical method

Statistical analysis of all data was performed using IBM spss19.0 software. The quantitative data was analyzed with descryiptive statistics.

## CONCLUSION

In conclusion, our study showed that the set-up errors were not consistent in all directions over the course of IMRT. The experience that the PTV designed from a certain margin that was uniformly expanded based on the CTV was not suitable for current precise radiotherapy technology. Further studies are required to establish the appropriate data for the CTV-PTV margins in IMRT planning.
